# Modelling the Influence of Task Constraints on Goal Kicking Performance in Australian Rules Football

**DOI:** 10.1186/s40798-021-00393-9

**Published:** 2022-01-24

**Authors:** Peter R. Browne, Alice J. Sweeting, Sam Robertson

**Affiliations:** 1 Institute for Health and Sport (iHeS), Victoria University, Ballarat Road, Footscray, VIC 3011 Australia; 2 Western Bulldogs, 417 Barkly Street, Footscray, VIC 3011 Australia

**Keywords:** Machine learning, Performance analysis, Constraints-led approach, Visualisations

## Abstract

**Background:**

The primary aim of this study was to determine the influence of task constraints, from an ecological perspective, on goal kicking performance in Australian football. The secondary aim was to compare the applicability of three analysis techniques; logistic regression, a rule induction approach and conditional inference trees to achieve the primary aim. In this study, an ecological perspective has been applied to explore the impact of task constraints on shots on goal in the Australian Football League, such as shot type, field location and pressure. Analytical techniques can increase the understanding of competition environments and the influence of constraints on skilled events. Differing analytical techniques can produce varying outputs styles which can impact the applicability of the technique. Logistic regression, Classification Based on Associations rules and conditional inference trees were conducted to determine constraint interaction and their influence on goal kicking, with both the accuracy and applicability of each approach assessed.

**Results:**

Each analysis technique had similar accuracy, ranging between 63.5% and 65.4%. For general play shots, the type of pressure and location particularly affected the likelihood of a shot being successful. Location was also a major influence on goal kicking performance from set shots.

**Conclusions:**

When different analytical methods display similar performance on a given problem, those should be prioritised which show the highest interpretability and an ability to guide decision-making in a manner similar to what is currently observed in the organisation.

## Key Points

- Constraints have varying influences and interaction with one another to influence skilled performance.
- Location is a major influence on goal kicking, however, understanding the other constraints present is also key to understanding performance objectively.
- The visualisation and reporting techniques used are a factor when deciding methodology to use for the uptake of analysis in the applied setting.

## Introduction

It is well established that sports performers are constantly exposed to numerous constraints that manifest both concurrently and continuously [1–3]. From an ecological perspective, constraints can influence a system and shape the emergence of functional movement solutions [1, 2, 4]. Constraints are commonly classified into individual, task and environmental categories [2]. This research focuses on task constraints which relate to the intent of the activity [5] and are more specific to competition performance compared with environmental constraints [1]. Constraints interact nonlinearly to influence skilled performance of both teams and individuals [6–8]. Typically, research in sport has tended to isolate one or two constraints as opposed to acknowledging these interactions [8]. This has potentially occurred due to many influential constraints having not been previously measurable in a sufficiently valid or reliable manner (i.e. available time for decision-making and psychological pressure). The identification of constraints, such as wind speed and direction, location and pressure can improve an understanding of the competition environment to better evaluate competition performance and inform training design [9]. However, improvements to technology have meant that many of these aspects are now feasibly measurable in many sports environments.

The move towards the use of analytical methods capable of describing the complexity inherent in constraint interactions in sports environments represents an ongoing challenge. This is despite many machine learning algorithms have the ability to account for nonlinear interactions of multiple variables (i.e. constraints) [10]. Furthermore, varying analytical techniques may enable a range of outputs and visualisations of constraint interactions. These consequently produce different opportunities for action by the end user, which may be more or less suitable depending on their intended purpose (i.e. training design, performance evaluation) or preferences. These outputs may influence how the findings can be presented and interpreted, along with the specific type of decision that is recommend (i.e. recommendation or prediction).

An analysis technique will most likely be implemented if its interpretability and functionality fit within the type of operational framework applied in that setting [11]. Therefore, the design and style of results are critical in guiding decision-making [12]. Some complexity can be reduced by translating information into visuals, thus reducing the cognitive work required to interpret written reports [13]. The application of findings may be supported by visualisations and increased practitioner education to have a positive impact in the sporting domain. Therefore, the accuracy of an analysis technique is not the only factor in the applied setting, but also the manner in which results are presented. Further, it could enable the incorporation of a more mainstream use of machine learning into performance evaluation in competition and training. Accordingly, this may facilitate a move away from a reductionist approach towards capturing and analysing multiple variables concurrently.

Australian Football (AF) is a complex invasion style sport, played on an oval (length =  ~ 160 m, width =  ~ 130 m). A goal, worth six points, is scored by kicking a ball through two upright middle posts at the team’s attacking end of the ground. A behind, worth one point, is scored by the ball going between the outer two posts [14, 15]. Accurate goal kicking has been identified as the most influential performance indicator of match outcome [16]. Within AF, goal kicking technique has been found to alter based on kick distance [17]. Despite this, limited research has explored how constraints interact to influence goal kicking performance in AF [18]. However, research in other team invasion sports has explored goal kicking or shooting. Within American Football, the role of perception has been explored in punt and goal kicking outcome [19]; in basketball shooting, accuracy has been explored through location [20, 21], movement variability [22] and defensive influence [23]; in soccer the impact of different conditions and ball size has been used to explore altered kinematics [24]. The interaction of constraints has also been explored in the Rugby Union place kicking and shown to influence performance [7].

The primary aim of this study was to determine the influence of task constraints on goal kicking performance in Australian football. The secondary aim was to compare the applicability of three analysis techniques; logistic regression, a rule induction approach and conditional inference trees to achieve the primary aim.

## Methods

**Fig. 1:**
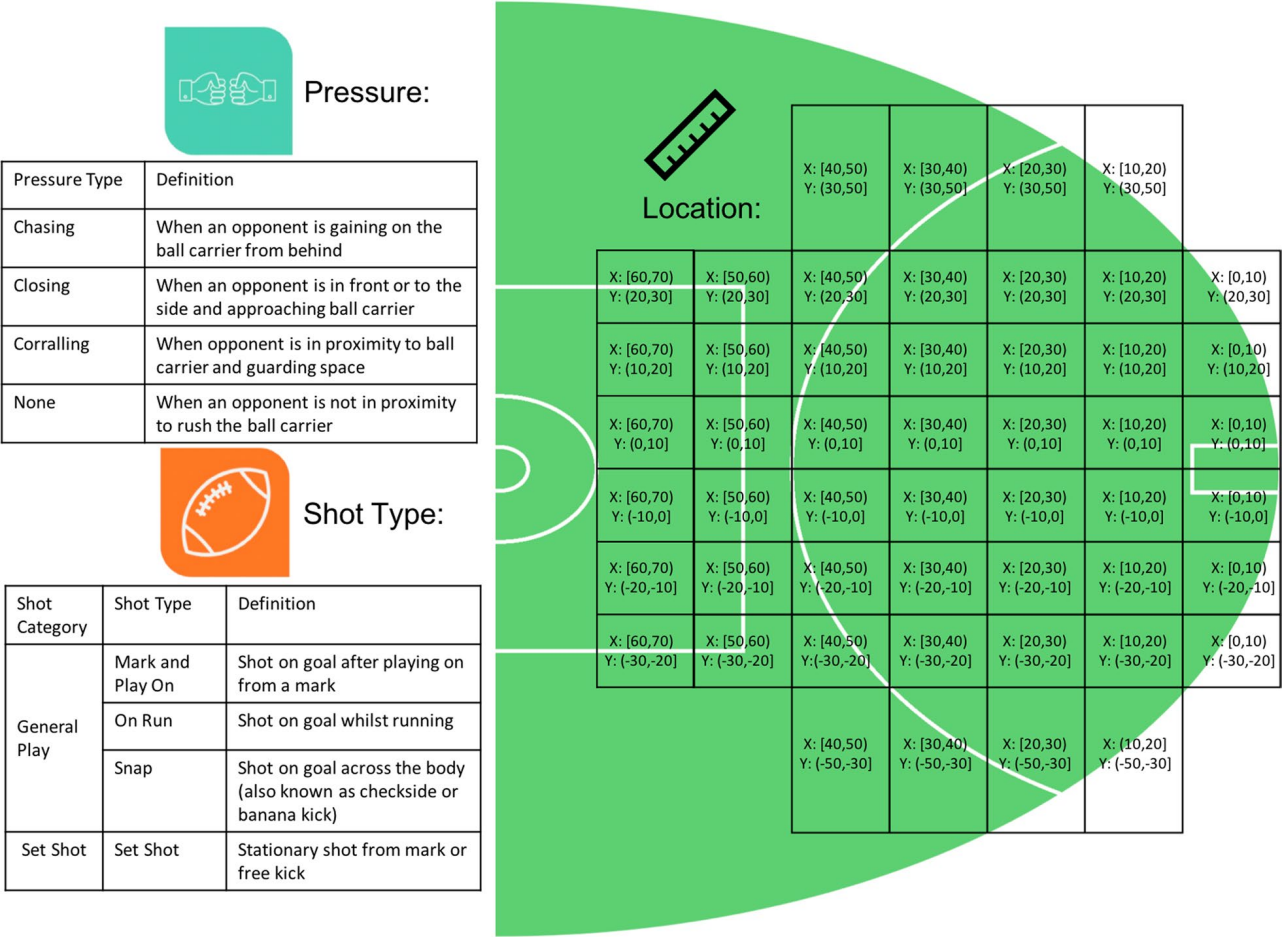
Breakdown of constraints for set and general play shots

**Fig. 2:**
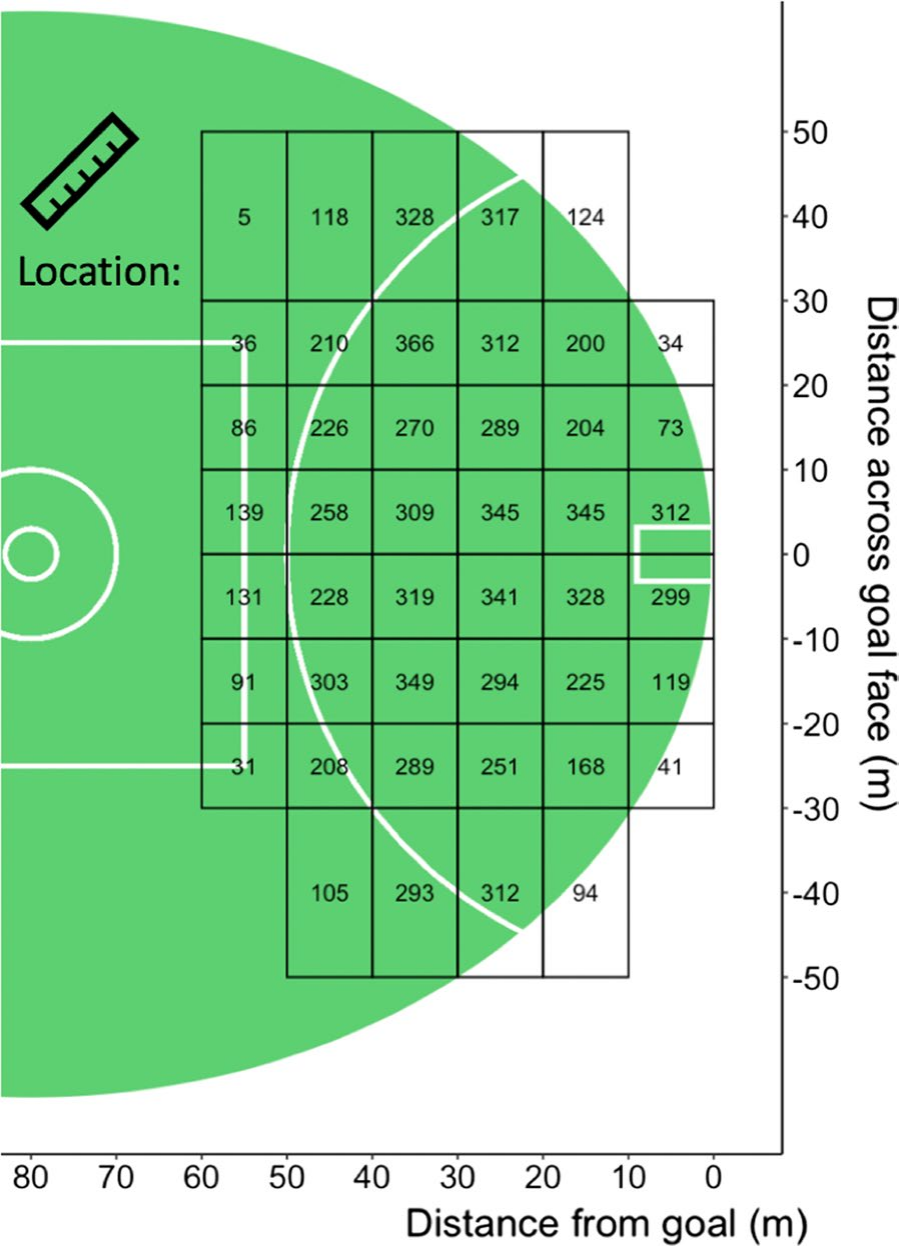
Number of shots from each location for entire dataset

Data were collected from all games (*n* = 207) conducted in the 2017 Australian Football League (AFL) season. Data were obtained from Champion Data, with permission provided for use in this study. Champion Data have not publicly released the validity and reliability of these data; however, research has found very high levels of agreement between Champion Data and independent evaluation [25]; this study did not conduct an independent evaluation of the accuracy of the data. All shots on goal contained additional information on shot type, shot outcome, pressure type and location. The pressure variable was manually collected by Champion Data based on the action and direction of opposing defender. Each variable had various sub-categories shown in Fig. 1. The constraints used in this study are included in the official statistics provided to AFL clubs and commonly used by AF teams in practice. Further have been shown to influence performance in team sport literature [18, 20, 26]. A total of 9725 attempted shots on goal were recorded; these were further split into set shots (*n* = 4939) and general play shots (*n* = 4786). The former refers to a goal shot in which the player has up to 30 s to attempt the kick without being actively defended [18], excluding where “play on” or “advantage” is called by the umpire. All other shots fit the category of general play. Further, shot location was divided into *X* (distance from goal) and *Y* (distance across the goal face). Distances were grouped in 10 m increments from 0 to 60 m in the *X* axis, and 10 m increments within 20 m across the goal face. Outside of 30 m, they were grouped in 20 m due to the infrequencies of shots in these ranges (Figs. 1, 2). Ethical approval was granted by the University Human Research Ethics Committee (application number: HRE18-022).

Three techniques, logistic regression, a rule induction approach and conditional inference trees were chosen as, whilst they each treat shot outcome as a classification problem, they each consider independent variables differently and produce different outputs. The three techniques were run separately for all shots, set shots and general play shots, resulting in the generation of nine models. For all models, data were split into an 80% training set and a 20% testing set. All analyses were conducted in R (Version 3.1.2, R Foundation for Statistical Computing, Vienna, Austria). Model performance was defined by mean accuracy (%) between test and training datasets. Confusion matrices were also produced and levels of precision, recall and F1 were calculated for each model. Precision informs how accurate a model is at determining true positives from actual results, whereas recall measures the fraction of true positives from the predicted results. The F1 metric provides measured balance between precision and recall. For further information on calculating these metrics, see Lipton et al. [27].

### Logistic Regression

Logistic regression is a mathematical modelling technique which is used to describe the relationship of several independent variables to a dependent variable [28]. This technique is widely used for the identification of variables which relate to sports performance, when working with a dichotomous dependent variables [28]. Logistic regression models considered the relationship between location, pressure and shot type constraints (independent variables) and the binary shot outcome, goal or no goal (dependent variable).

### Classification Based on Association Rules (CBA)

Rule induction is a branch of machine learning, capable of identifying underlying and frequent patterns between variables in a large transactional database [8, 29–31]. The *Classification Based on Association rules* (CBA) algorithm is an unsupervised data mining technique [32]. The CBA algorithm was run in R, using the *‘arulesCBA’* package [33]. A shot on goal was treated as the ‘transaction’, with the dependent variable specified as goal or no goal, and the constraints were used to describe it. Rules generated by the CBA algorithm were measured by their levels of *Support* and *Confidence,* see Eqs. 1 and 2. Minimum support and confidence were set at 0.005 to allow for rules to be generated for each location bin where a shot took place. Where the minimum criteria were not met, no rule was generated and the output for that location was left blank. Outputs were limited to rules containing all relevant constraints for the set shot and general play model.

1 $${\text{support}}\left( {A = > B} \right) = P\left( {A \cup B} \right)$$

2 $${\text{confidence}}\left( {A = > B} \right) = \frac{{{\text{support}}\left( {A \cup B} \right)}}{{{\text{support}}\left( A \right)}} = P\left( {B|A} \right)$$

### Conditional Inference Trees

Conditional inference trees provide another nonlinear approach to quantify the relationship between dependent variables [34]. They are a supervised machine learning technique which consist of a range of significance tests to determine a threshold for each dependent variable [34, 35]. Branches consist of a different combination of response variables, shot outcome, which leads to the prediction of the independent variable [34]. Conditional inference trees were generated using the *party* package in R [36]. The algorithm functions on a predetermined level of statistical significance (*p* < 0.05), and factors which are most strongly linked with the response variable (goal or no goal) underwent recursive partitioning [34, 35]. Each tree was developed with a 95% confidence interval (CI) under a Bonferroni correction and a minimum terminal node size of 400 instances. The first tree was developed on the set shot dataset utilising two parameters, *X* and *Y* location. The second was run with the general play dataset and included four parameters, all constraint variables.

## Results

**Fig. 3:**
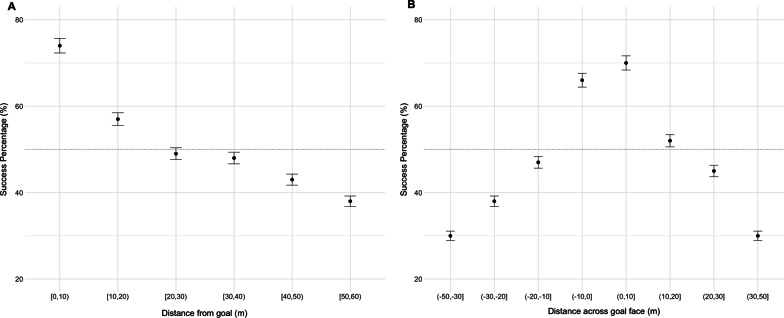
The mean success of shots on goal by location. Distance from the goal line in length (**A**) and distance across the goal face in width (**B**), where 0 is the centre of the goal. Confidence interval of 95% is shown. The horizontal dotted line represents the average success rate of a shot on goal

Of the shots on goal attempted in the 2017 AFL season, mean shot accuracy was 50.4%. To understand how distance solely influenced shot success, the odds of success at each distance were calculated for width and length from the goal face (Fig. 3).

The three models showed similar levels of mean accuracy for all shots. Differences existed in levels of recall, precision and F1 (Table 1). The CBA model had the highest level of recall (0.73); however, this was a trade-off given the low level of precision (0.40), whereas the logistic regression and conditional inference models showed a slightly lower recall value, 0.62 and 0.68, respectively, the precision value was higher at 0.85 for both, which resulted in a greater F1 value, 0.72 and 0.76, respectively (see Table 1).

**Table 1:** Confusion matrix and derived metrics for all shots in test dataset for each model

*n* = 1941	Actual					
	Logistic regression		CBA		Conditional inference	
	Goal	No goal	Goal	No goal	Goal	No goal
*Predicted*						
Goal	846	146	383	566	491	458
No Goal	526	423	143	849	232	760
Model mean accuracy	65.4%		63.5%		64.5%	
Recall	0.62		0.73		0.68	
Precision	0.85		0.40		0.85	
*F*1	0.72		0.52		0.76	

### Logistic Regression

The logistic regression model predicted shot outcome in the test data set at 65.4% for all shots, 64.9% for set shots and 67.2% for general play shots. Both independent variables of *X* and *Y* locations impacted the outcome of set and general play shots. The four independent variables: *X*, *Y*, shot type and pressure were all correlated with the outcome of all shots based on the odds ratio (Table 2). For set shots, only *X*—(10,20] had an odds ratio of less than one, 0.95. For general play shots, two variables had an odds ratio of less than one, a pressure level of none and *Y*—(0,10], 0.81 and 0.93, respectively (Table 2).

**Table 2:** Logistic regression coefficients for set and general play shots

	Coefficients	Estimate	SE	*z* Value	Odds ratio	CI.95	*p* Value
Set shots	(Intercept)	− 1.66	0.19	− 8.55			
	X—[0,10)	Ref					
	X—[10,20)	− 0.05	0.20	− 0.26	0.95	(0.65,1.40)	0.8
	X—[20,30)	0.01	0.19	0.03	1.01	(0.70,1.45)	0.97
	X—[30,40)	0.24	0.18	1.32	1.28	(0.89,1.83)	0.19
	X—[40,50)	0.81	0.19	4.38	2.25	(1.57,3.24)	< 0.01
	X—[50,60)	1.54	0.23	6.74	4.66	(2.98,7.28)	< 0.01
	Y—(− 10,0]	Ref					
	Y—(− 20, − 10]	1.03	0.14	7.30	2.81	(2.13,3.71)	< 0.01
	Y—(− 30, − 20]	1.60	0.15	10.62	4.97	(3.70,6.69)	< 0.01
	Y—(− 50, − 30]	2.19	0.15	14.27	8.98	(6.64,12.14)	< 0.01
	Y—(0,10]	0.02	0.15	0.14	1.02	(0.76,1.37)	0.89
	Y—(10,20]	1.00	0.15	6.77	2.71	(2.03,3.61)	< 0.01
	Y—(20,30]	1.44	0.14	10.08	4.23	(3.20,5.61)	< 0.01
	Y—(30,50]	2.04	0.15	13.72	7.67	(5.74,10.27)	< 0.01
General play shots	(Intercept)	− 2.35	0.23	10.12			
	X—[0,10)	Ref					
	X—[10,20)	0.47	0.14	3.42	1.60	(1.22,2.09)	< 0.01
	X—[20,30)	0.90	0.13	6.79	2.47	(1.90,3.20)	< 0.01
	X—[30,40)	1.05	0.14	7.76	2.87	(2.20,3.74)	< 0.01
	X—[40,50)	1.52	0.16	9.79	4.59	(3.38,6.22)	< 0.01
	X—[50,60)	1.87	0.19	9.83	6.48	(4.47,9.41)	< 0.01
	Y—(− 10,0]	Ref					
	Y—(− 20, − 10]	0.74	0.12	6.21	2.09	(1.66,2.64)	< 0.01
	Y—(− 30, − 20]	1.31	0.14	9.15	3.70	(2.79,4.89)	< 0.01
	Y—(− 50, − 30]	1.70	0.20	8.61	5.47	(3.71,8.05)	< 0.01
	Y—(0,10]	− 0.08	0.11	− 0.69	0.93	(0.75,1.15)	0.49
	Y—(10,20]	0.34	0.12	2.74	1.40	(1.10,1.78)	< 0.01
	Y—(20,30]	0.98	0.13	7.41	2.65	(2.05,3.43)	< 0.01
	Y—(30,50]	1.84	0.18	9.97	6.32	(4.40,9.08)	< 0.01
	Mark Play On	Ref					
	On Run	0.60	0.16	3.72	1.83	(1.33,2.51)	< 0.01
	Snap	1.21	0.17	7.14	3.34	(2.40,4.65)	< 0.01
	Pressure-chasing	Ref					
	Pressure-closing	0.55	0.16	3.50	1.74	(1.28,2.37)	< 0.01
	Pressure-corralling	0.14	0.14	0.95	1.15	(0.86,1.52)	0.34
	Pressure-none	− 0.22	0.16	− 1.37	0.81	(0.59,1.10)	0.17
	Pressure-physical	1.23	0.19	6.50	3.43	(2.36,4.97)	< 0.01

### Classification Based on Association Rules (CBA)

The accuracy of the CBA model was 63.5% for all shots with an F1 of 0.52, and mean model accuracy for 63.8% for set shots and 63.3% for general play shots. The CBA algorithm produced differing numbers of rules which met the set criteria depending on the contextual variables selected. Confidence levels ranged from 0.18 to 0.99.

### Conditional Inference Trees

Conditional inference trees predicted shot outcome with an *F*1 value of 0.76 and mean model accuracy of 64.5% for all shots, 64.7% for set shots and 64. 2% for general play shots. It revealed both *X* and *Y* locations to be strong indicators of shot success (Fig. 6). For set shots, the first partition was displacement in *Y* axis. The second partition was a further divide in the *Y* axis and displacement in *X* axis (Fig. 6). The third and final level of partition was in the *X* and *Y* axis.

General play shots on goal also revealed that all independent variables were important factors in predicting shot outcome. The tree’s first partition included *Y* displacement, distance from goal, and *X* and *Y* displacement formed the second split, and the final partition formed by *X* displacement or alternatively by pressure level (Fig. 7). Shot type did not form a split.

## Discussion

This study aimed to determine the influence of task constraints on goal kicking performance in Australian football. The study also compared the applicability of three analysis techniques: logistic regression, CBA and conditional inference trees. The different analysis techniques had similar accuracy levels. Given the similar performance of the different analysis techniques, consideration of their levels of applicability and ability to guide different types of decision-making should guide their respective use in the applied setting.

**Fig. 4:**
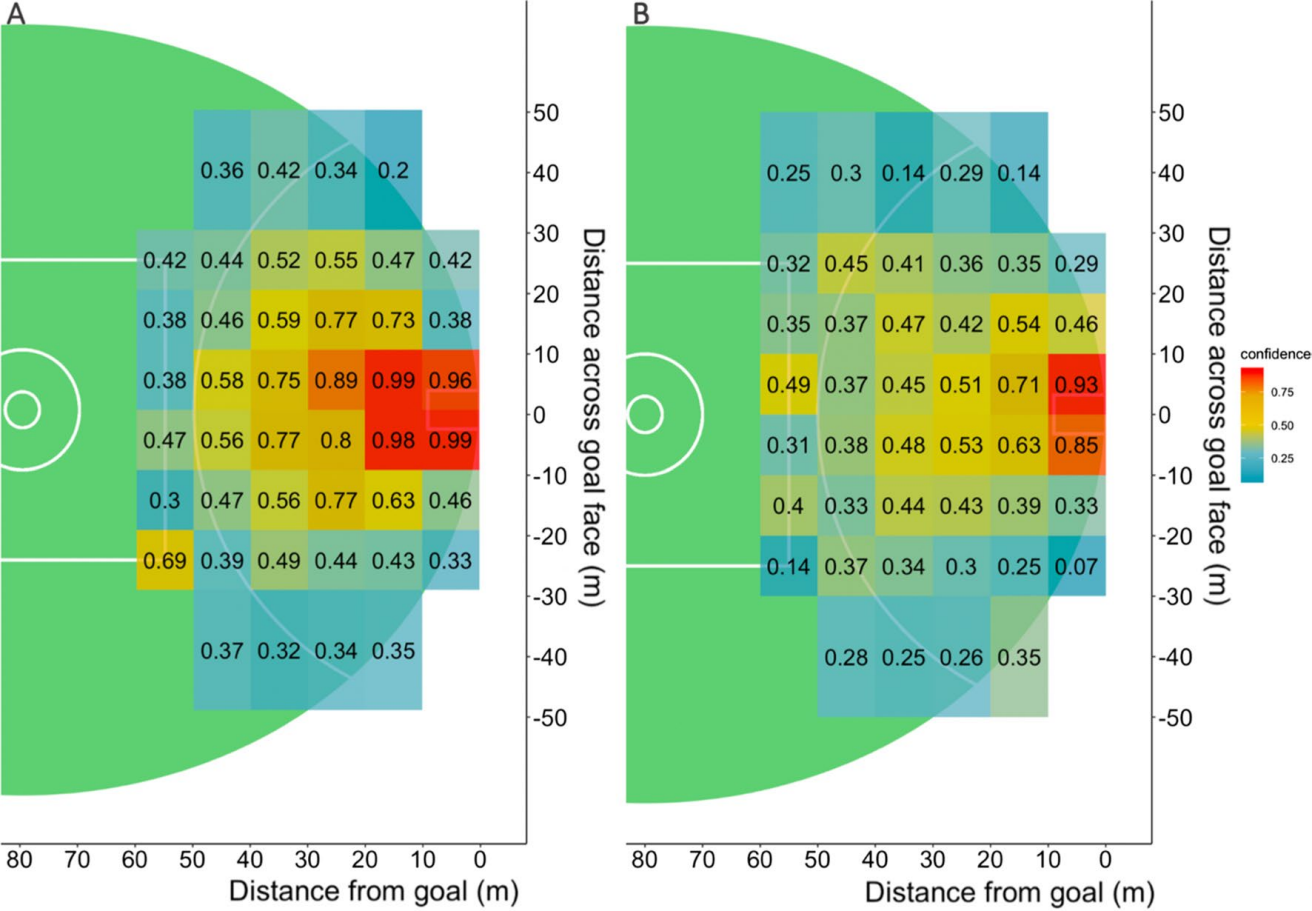
Confidence levels for each bin based on CBA outputs. **A** Set shots, **B** general play shots where the minimum criteria were met

**Fig. 5:**
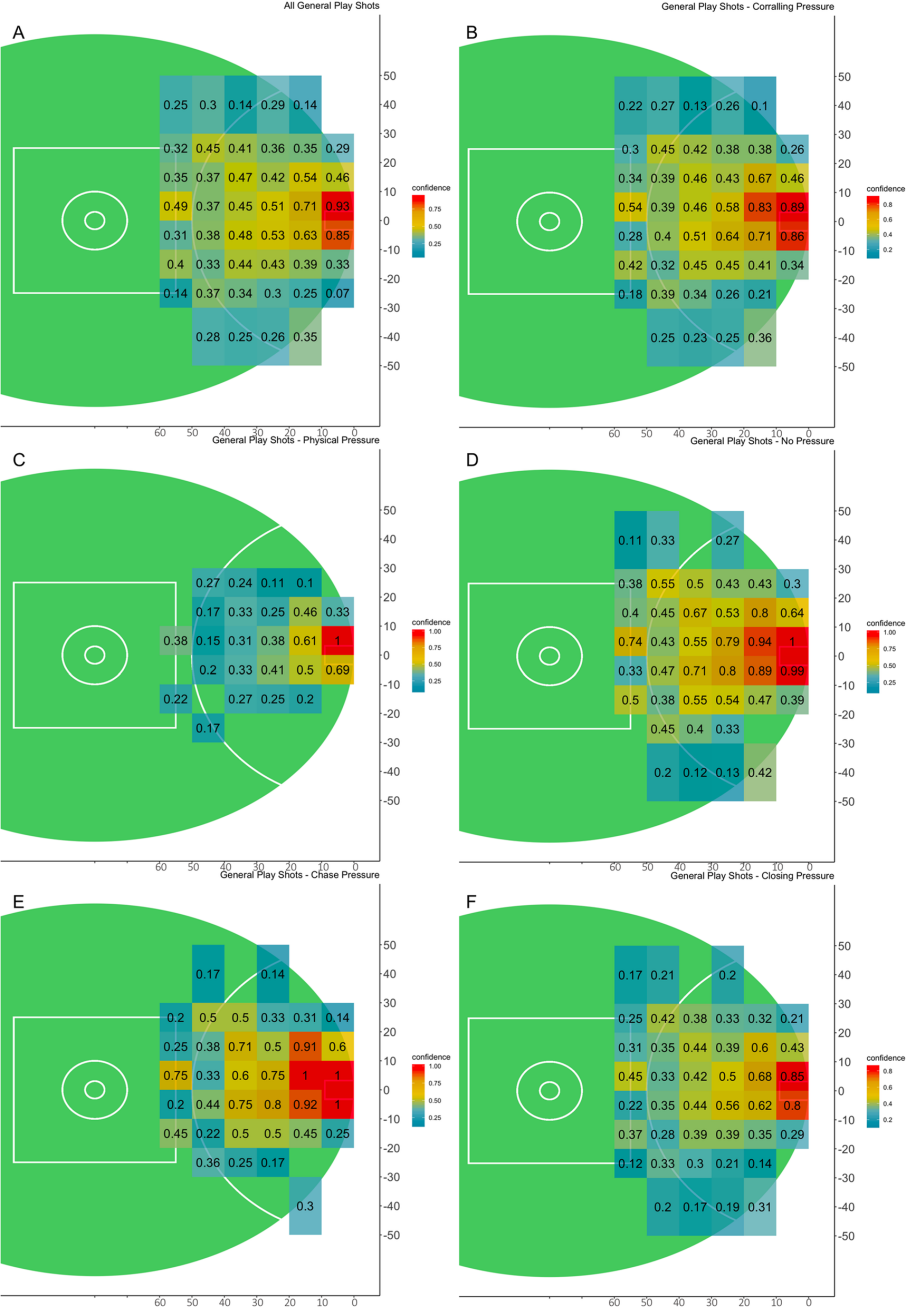
Confidence levels for each bin based on different pressure types. **A** All general play shots, **B** shots under corralling pressure, **C** shots under physical pressure, **D** shot with no pressure, **E** shots under chase pressure, **F** shots under closing pressure, where the minimum criteria were met

**Fig. 6:**
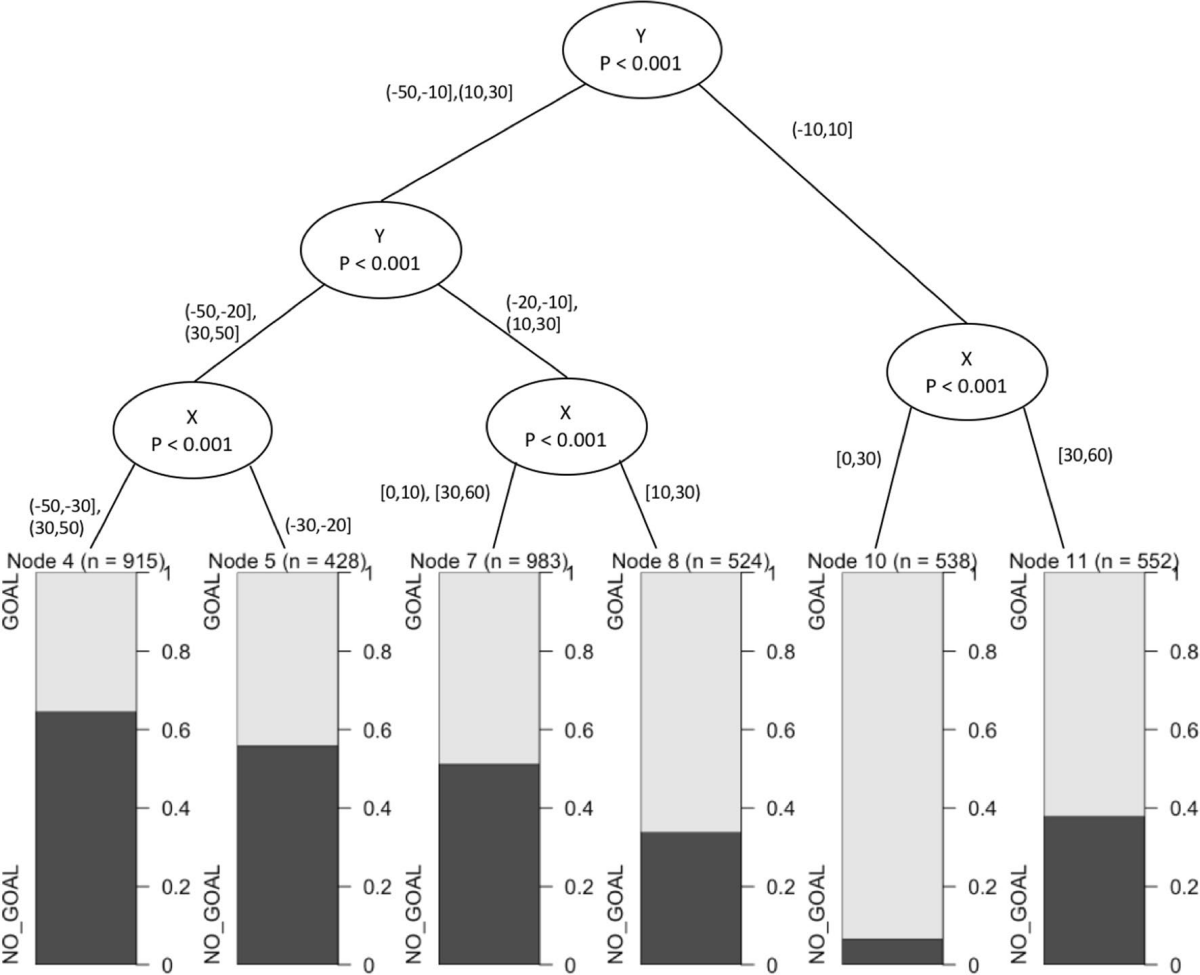
Conditional inference tree for set shots. Location (*X* and *Y* axis) as the independent variable and shot outcome as the dependent variable

**Fig. 7:**
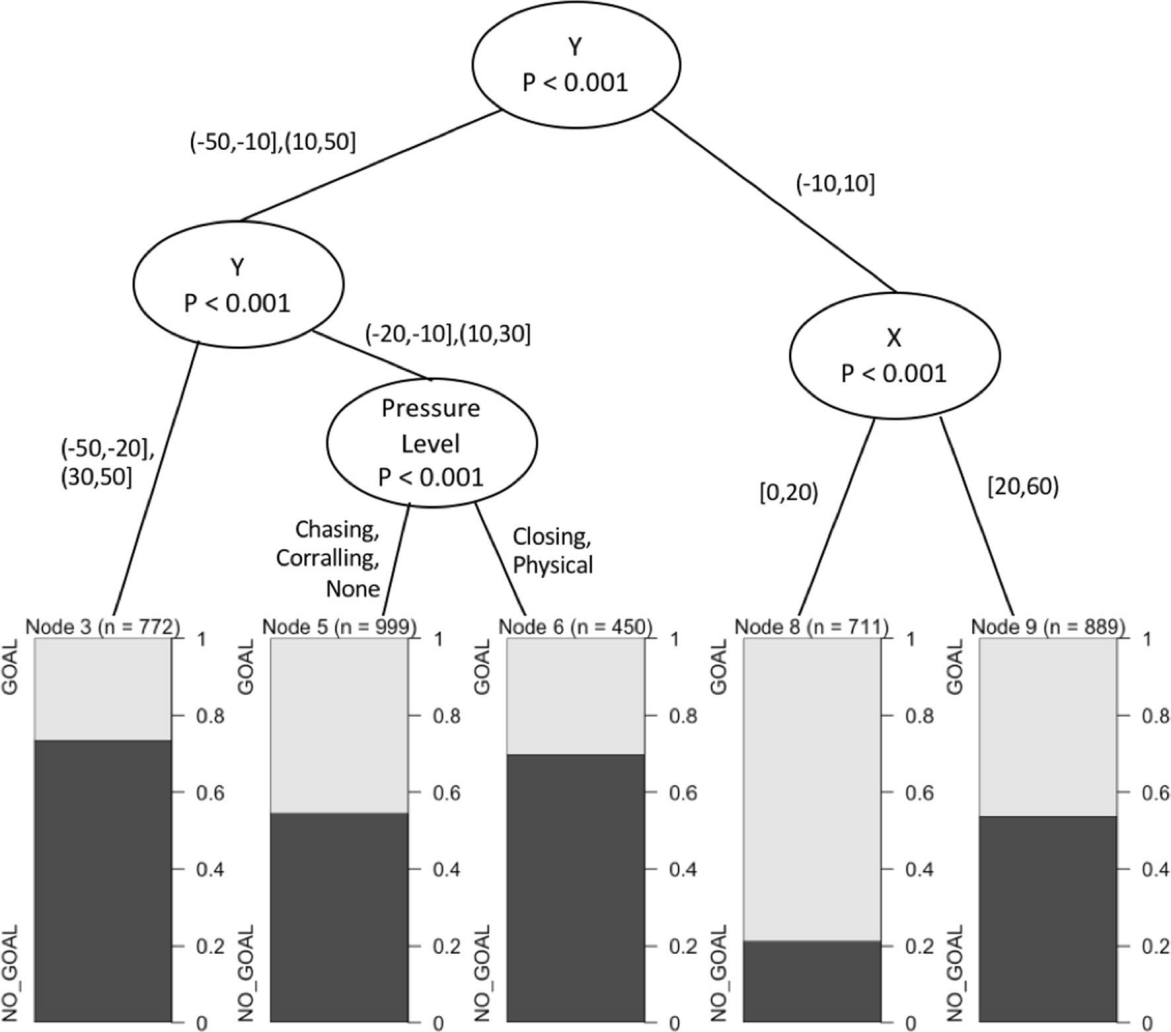
Conditional inference tree for general play shots. Location (*X* and *Y*), pressure type and shot type as the independent variables and shot outcome as the dependent variable

The constraints of location, pressure and shot type all influenced shot outcome. The likelihood of scoring altered as location changed, this was demonstrated across each analysis techniques applied (Table 2, Figs. 2, 4, 5, 6, 7). A potential explanation for this influence is the change of technique based on kick distance [17]. These findings align with research in other invasion sports, where location has also been identified as a predictor of kicking success in Rugby Union [7]. Further, two types of pressure, corralling and none, had the least influence on goal kicking accuracy in the logistic regression model (Table 2). Both the logistic regression and conditional inference trees showed closing and physical pressure to have a negative impact on shot outcome (Fig. 7). Location and defensive pressure have been shown to influence shot outcome in basketball [21, 23]. Shot type did not create a branch within the general play conditional inference tree (Fig. 7). However, differences between set shot and general plays shot success are demonstrated (Figs. 4, 6, 7).

Areas of equal opportunity in AF goal kicking success have been calculated in AF [37]. These should not only be calculated by location, but also include additional constraints such as physical pressure and shot type. The CBA and conditional inference trees (Figs. 5, 6, 7) demonstrated the similarity between combinations of constraint types and likelihood of goal kicking accuracy. Areas of equal opportunity are shown in Fig. 5C, D where a shot under physical pressure from *X*(0,10) and *Y*(20,30) has a confidence level of 0.38 in contrast with a shot under no pressure from *X*(40,50) and *Y*(20,30) which has a confidence level of 0.55. This is also demonstrated by the CBA as a general play shot taken from the same location has a very different likelihood of a goal based on the type of pressure (Fig. 6). This demonstrates how it may be beneficial to move the ball wider and further from goal, to avoid taking a shot under physical pressure. This information could be applied to inform decision-making around shot selection and education around the concept of areas of equal opportunity so that players understand that simply being closer to goal does not increase the likelihood of scoring, but the context of pressure and the shot type available will impact the outcome, thus this style of analysis provides a potential educational tool for athletes and coaches.

The analysis techniques utilised in this study displayed similar levels of accuracy and present the influence of constraints differently. The logistic regression model was more accurate at predicting a goal, whereas in contrast, the CBA and conditional inference models were better at predicting no goal (Table 1). For the logistic regression models, the influence of different constraints is evident, such as the impact of experiencing physical pressure compared with no pressure (Table 2); however, the interaction between constraints is difficult to observe. For instance, this model displays constraints independently and any interactions cannot be explored nonlinearly like in other methods. In contrast, the CBA and conditional inference trees models permit nonlinear interaction of constraints and their combined influence on goal kicking as a part of their inherent design as demonstrated (Figs. 4, 5, 6, 7). Whilst, each analysis technique suggests similar the patterns of constraint influence how these results and interaction is visualised varies.

The applicability of an analysis technique is due to more than its accuracy, but also how easily it can be interpreted and implemented. Benefits of the CBA and conditional inference tree techniques are both their nonlinear nature and visual output. These have the potential to demonstrate the interaction of multiple constraints. For example, a snap shot at goal is more likely based on a location close to the boundary line, whereas a shot from 50 m from goal whilst under chasing pressure would be a drop punt kick on the run. This may inform the design of goal kicking drills that better replicate competition, as they can consider both the frequency and prevalence of constraints. Dadzie and Rowe [38] suggest that visualisations may enhance the understanding of data, leading to the ability to enable instinctive and effective knowledge discovery. This is partly due to the decreased cognitive work required to interpret visualisations, as visuals take advantage of innate human perception [13, 38, 39]. In this, whilst the accuracy of each technique in determining the outcome of a shot on goal was similar, the CBA and conditional inference tree techniques may have an advantage over the logistic regression in regards to applicability, clear visuals may aid in-game and post-game assessment of shot selection and execution, although this was not formally investigated in this study.

Utilising multiple analysis techniques allows for the demonstration of variation and importance in model outputs and visuals. This is critical as findings may not be always be interpreted accurately and used effectively to inform decision-making [40, 41]. Further, when providing results to coaches, their willingness to accept and apply findings is critical [42]. Thus, a less accurate model, such as CBA which had the lowest F1 value, may be utilised over a slightly more accurate technique, due to the reduced complexity and higher interpretability of the model output. If results are too complex to interpret, then the likelihood of the findings being implemented is minimal. Thus, multiple analysis techniques can provide benefit in offering varied options to display results which can be aligned to the individual users. For instance, for some coaches, an understanding of how each individual constraint influences a shot on goal may help narrow down focus areas within training drills or guide language cues. Yet, other visualisations such as the heat map style of the CBA may enable a different perspective of potential kick success. They may help a coach understand how a team or individual is performing under certain circumstances in time-restricted environments. For example, in competition, being able to quickly understand how a shot success is being influenced by multiple constraints may help with decision-making in regards to personnel changes, tactics or messaging to players. Furthermore, if coaches are able to see differences in outcomes for kicks under the same constraints in training and competition settings, it may help to better inform drill design [43, 44]. The conditional inference tree visualisation provides a clearer grouping of similar opportunities and may aid coaches in educating athletes around their decision-making and what shot opportunities have an increased likelihood of success. For instance, a player may be passing the ball off to someone they think is in a better location to shoot from; however, they may be equally likely to be successful, therefore, they should take the shot themselves and not increase the chance of a turnover by making an additional pass. Ultimately, a coach may have a preference on how data are presented and being able to visualise multiple techniques allows for the customised presentation of results to suit coaches needs at a given time. Using the examples above, a coach may prefer a heatmap during a game or in post-game reporting to quickly demonstrate how the team performed under given match, whereas they may prefer to use a tree-like visual to further understand areas of equal opportunity to help develop an attacking game plan and team structures during planning sessions. It has also been suggested that appropriate staff should be embedded within professional clubs to aid in the statistical interpretation and applicability in industry settings, however, producing analysis in practitioner friendly formats is also of use [45].

Future research could include additional constraints within the models. Examples of additional constraints may include, exploring the game context such as time remaining and score margin as well as individual traits such as playing position and preferred foot [7, 46]. Additional data and the identification of key constraints which influence goal kicking could lead to more accurate models. This may help improve model accuracy to levels to make appropriate inferences from these data. Further data would enable the field to be divided into smaller regions to create more specific findings, as well as the potential to develop individual or team specific models. This would have a major impact in improving the accuracy and applicability of each model. Improved data capture may reduce subjectivity which currently exists in the measurement of currently collected constraints (for example see, Behendi et al. [47], Nibali et al. [48] and Victor et al. [49]). For instance, a constraint such as pressure could be measured on a continuous scale or as via a density metric [50].

## Conclusion

This study showed the influence of task constraints on goal kicking performance in AF, with differences between both set shots and general play shots accuracy based on location and pressure type. Of the three analysis techniques utilised, each has different benefits, for instance the logistic regression explored each constraint individually and the independent influence of constraints is clear. Contrastingly, CBA and conditional inference trees can aid in identifying nonlinear patterns more easily due to the ability to quickly visualise how multiple constraints interact together to influence shot outcome. Using the same dataset with different analysis techniques allows for varying outputs and visuals which demonstrate differences in the applicability of each model for the applied setting. Ultimately, preferences will come down to the individual user. This information may further the understanding of competition conditions to enhance training design and enable the better preparation of players to meet competition demands.

## Data Availability

The code used in this study is available from the corresponding author on reasonable request. The data that support the findings of this study are available from Champion Data but restrictions apply to the availability of these data, which were used under license for the current study, and so are not publicly available. Data are however available from the authors upon reasonable request and with permission of Champion Data.

## Abbreviations

AF: Australian football
AFL: Australian football league
CBA: Classification based on association rules
CI: Confidence interval
